# Molecular detection of fungal pathogens in clinical specimens by 18S rDNA high-throughput screening in comparison to ITS PCR and culture

**DOI:** 10.1038/s41598-018-25129-w

**Published:** 2018-05-03

**Authors:** K. Wagner, B. Springer, V. P. Pires, P. M. Keller

**Affiliations:** 1 Institute of Medical Microbiology, University of Zurich, Zurich, Switzerland; 2 Institute of Medical Microbiology and Hygiene, Austrian Agency for Health and Food Safety, Graz, Austria

## Abstract

The rising incidence of invasive fungal infections and the expanding spectrum of fungal pathogens makes early and accurate identification of the causative pathogen a daunting task. Diagnostics using molecular markers enable rapid identification of fungi, offer new insights into infectious disease dynamics, and open new possibilities for infectious disease control and prevention. We performed a retrospective study using clinical specimens (N = 233) from patients with suspected fungal infection previously subjected to culture and/or internal transcribed spacer (ITS) PCR. We used these specimens to evaluate a high-throughput screening method for fungal detection using automated DNA extraction (QIASymphony), fungal ribosomal small subunit (18S) rDNA RT-PCR and amplicon sequencing. Fungal sequences were compared with sequences from the curated, commercially available SmartGene IDNS database for pathogen identification. Concordance between 18S rDNA RT-PCR and culture results was 91%, and congruence between 18S rDNA RT-PCR and ITS PCR results was 94%. In addition, 18S rDNA RT-PCR and Sanger sequencing detected fungal pathogens in culture negative (N = 13) and ITS PCR negative specimens (N = 12) from patients with a clinically confirmed fungal infection. Our results support the use of the 18S rDNA RT-PCR diagnostic workflow for rapid and accurate identification of fungal pathogens in clinical specimens.

## Introduction

In recent years an enormous increase in the frequency and severity of fungal infections has been observed^1,2^. Medical progress has also contributed to increased opportunistic infections in patients, who are immunocompromised or who are infected during intensive care treatment^3^. Rapid diagnosis of fungal infections is a key factor for patient outcome^4^. Traditionally, diagnosis of fungal infection has relied primarily on direct microscopic examination of clinical samples, histopathology and culture^5,6^. However, diagnostic performance of microscopic examination and culture are highly dependent on the quality of the clinical specimens and the experience of the laboratory personnel. Moreover, these classical methods have previously shown lower sensitivity in fungal detection compared to molecular methods^7–9^. PCR based assays do not require viable cells and have the power to identify the continuously increasing number of fungi that are clinically relevant pathogens in humans, including rarely encountered species^9,10^. Fungal ribosomal genes are multicopy targets, facilitating an efficient detection using PCR, while the conserved nature of the 18S rRNA gene enables accurate sequence identification^11,12^. One limitation for the evaluation of new diagnostic methods remains the lack of a gold standard as culture and microscopic examination have been proven to be less sensitive than PCR based methods^7–9,13^. Therefore, a more appropriate approach is a compilation of clinical and microbiological data as gold standard to evaluate the diagnostic performance of new molecular methods.

**Figure 1:**
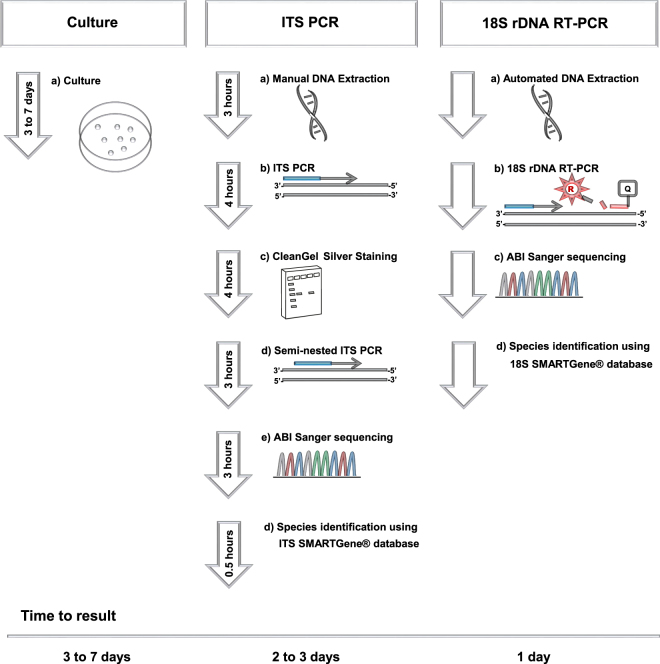
Overview of the diagnostic algorithms used for identification of fungal pathogens at the Institute of Medical Microbiology, University of Zurich.

In this retrospective study, we used clinical specimens that have been previously analysed by culture and/or internal transcribed spacer (ITS) PCR and could be categorized as “confirmed fungal infection” or “no fungal infection”. These specimens were retrospectively analysed by RT-PCR amplification of the fungal ribosomal small subunit (18S) and amplicon sequencing to rapidly and accurately identify fungal pathogens (Fig. 1).

## Results

### Diagnostic performance of 18S rDNA RT-PCR compared to ITS PCR

In total, 233 clinical samples were retrospectively analyzed, of those 143 specimens (61%) concordantly tested negative and 76 specimens (33%) tested positive by ITS and 18S rDNA sequencing. Fourteen of 233 clinical specimens (6%) showed discrepant results between both methods (Tables 1 and 2).

**Table 1:** Comparison of fungal identification by 18S rDNA RT-PCR and ITS PCR (N = 233) applying a gold standard that combines clinical and microbiologal data.

		clinical and microbiological data	
		positive	negative
18S rDNA RT-PCR	positive	88^a^	0
	negative	2	143
ITS PCR	positive	78^a^	0
	negative	12	143

^a^76 clinical specimens were congruently tested positive by both methods.

**Table 2:** Clinical specimens (N = 14) that showed discrepant results in fungal identification by ITS PCR and 18S rDNA RT-PCR.

sample number	clinical specimens	direct microscopic examination	species identification using culture	species identification using ITS PCR	Ct value in 18S rDNA RT-PCR	Species identification using 18S rDNA RT-PCR	clinical information
1	biopsy	nd	nd	negative	32.2	*Aspergillus* sp.	clinical suspicion of invasive aspergillosis
2	cornea	negative	negative	negative	34.3	*Fusarium* sp.	keratitis, clinical suspicion of invasive mycosis
3	paraffin section from epipharynx	nd	nd	negative	34.0	*Malassezia* sp.	osteomyelitis, invasive mycosis
4	paraffin section from sinus maxillaris	nd	nd	negative	33.3	*Malassezia* sp.	osteomyelitis, invasive mycosis
5	paraffin section from the upper lobe of the lung	nd	nd	negative	35.1	*Rhizopus* sp.	pulmonary mycetoma, clinical suspicion of invasive mucormycosis
6	paraffin section from epipharynx	positive	nd	negative	34.0	*Rhizopus* sp.	pulmonary zygomycosis, clinical suspicion of mucormycosis
7	biopsy from the sinus	positive	negative	negative	31.8	*Aspergillus* sp.	chronic fungal sinusitis, clinical suspicion of invasive aspergillosis
8	cornea	negative	negative	negative	31.1	*Fusarium* sp.	keratitis, clinical suspicion of invasive mycosis
9	blood (EDTA)	nd	negative	negative	30.0	*Rhizomucor pusillus*	clinical suspicion of invasive mycosis
10	blood (Citrate)	nd	negative	negative	33.6	*Cryptococcus* sp.	clinical suspicion of invasive mycosis
11	bronchoalveolar lavage	negative	negative	negative	34.8	*Pneumocystis jirovecii*	clinical suspicion of pneumocystis pneumonia in immunocompromised patient
12	paraffin section from the lung	nd	negative	negative	32.9	*Rhizomucor pusillus*	invasive mucormycosis of the lung
13	bronchoalveolar lavage	negative	negative	*Aspergillus* sp.	37.0	negative	clinical suspicion of invasive aspergillosis
14	abscess	negative	negative	*Candida albicans*	36.8	negative	abscess, invasive mycosis

nd = not done.

Sixty-five of 233 clinical specimens revealed no cycling threshold (Ct) value in the 18S rDNA RT-PCR and were therefore assessed negative. 168/233 showed amplification with a Ct value in the 18S rDNA RT-PCR. In 14 of those specimens, known environmental contaminants were identified (e.g *Funneliformis mosseae*, *Stereocaulon urceolatum*; Table S1), 64 specimens showed no fungal identification (i.e weak peaks or multiple overlying peaks in the Sanger electropherograms), and in 88 specimens a fungal pathogen was identified. In 62/233 specimens, no distinct ITS PCR fragment was visible in the CleanGel, and these specimens were assessed negative. 171/233 specimens showed an ITS PCR amplification product in the CleanGel. In 81 of those specimens, fungal identification was not possible or revealed known environmental contaminants, while in 78 specimens fungal pathogens were identified.

A diverse range of fungal genera were concordantly identified by ITS PCR and 18S rDNA RT-PCR, including *Aspergillus*, *Candida*, *Cryptococcus*, *Fusarium*, *Lichtheimia, Penicillium*, *Scedosporium, Rhizomucor* and *Rhizopus*. The five most prevalent fungal genera identified were *Candida* spp. (46%), *Aspergillus* spp. (18%), *Rhizomucor* spp. (7%), *Fusarium* spp. (5%) and *Lichtheimia* spp. (4%).

Out of 90 clinical specimens, in which fungal pathogens were present, ITS sequence analysis revealed species identifications in 59 specimens and led to identification on genus level in 19 specimens. Twelve samples did not show any relevant fungal identification by ITS sequencing (Table 2) and were therefore assessed negative. In contrast, 18S rDNA sequencing revealed 13 species identifications and 75 genus identifications. Two samples did not show any relevant fungal identification by 18S rDNA sequencing (Table 2) and were assessed negative.

Overall, the concordance of 18S rDNA RT-PCR and ITS PCR results for the diagnosis of fungal infections was 94% (Table 1). Sensitivity of the 18S rDNA RT-PCR was higher compared to ITS PCR, while specificity was equal among both methods using a composite diagnostic measure combining clinical and microbiological data as gold standard.

### Diagnostic performance of 18S rDNA RT-PCR compared to culture

Traditionally, conventional culture is used for the microbiological diagnosis of fungal infections. For 151/233 clinical specimens culture and 18S rDNA RT-PCR results were available. 104/151 specimens (69%) concordantly tested negative, and 34 specimens (22%) tested positive by culture and 18S rDNA sequencing. Thirteen of 151 (9%) clinical specimens showed discrepant results between both methods (Tables 3 and 4).

**Table 3:** Comparison of fungal identification by 18S rDNA RT-PCR and culture (N = 151) applying a gold standard that combines clinical and microbiologal data.

		clinical and microbiological data	
		positive	negative
18S rDNA RT-PCR	positive	47	0
	negative	0	104
Culture	positive	34	0
	negative	13	104

**Table 4:** Clinical specimens (N = 13) that showed discrepant results in fungal identification by culture and 18S rDNA RT-PCR.

no	clinical specimens	direct microscopic examination	species identification using culture	species identification using ITS PCR	Ct value in 18S rDNA RT-PCR	species identification using 18S rDNA RT-PCR	clinical information
1	biopsy from sinus maxillaris	positive	negative	*Aspergillus fumigatus*	32.0	*Aspergillus* sp.	chronic fungal sinusitis, clinical suspicion of invasive aspergillosis
2	wound secretion	positive	negative	*Aspergillus fumigatus*	31.7	*Aspergillus* sp.	abscess in the nose, clinical suspicion of invasive aspergillosis
3	wound secretion	negative	negative	*Aspergillus fumigatus*	30.6	*Aspergillus* sp.	chronic fungal sinusitis, clinical suspicion of invasive aspergillosis
4	biopsy from sinus maxillaris	positive	negative	*Aspergillus fumigatus*	30.0	*Aspergillus* sp.	chronic fungal sinusitis, clinical suspicion of invasive aspergillosis
5	deep wound	negative	negative	*Candida albicans*	29.6	*Candida* sp.	endograft infection after EVAR
6	biopsy from sinus	negative	negative	*Lichtheimia corymbifera*	26.6	*Lichtheimia corymbifera*	chronic fungal sinusitis
7	deep wound of the skin	negative	negative	*Malassezia sympodialis*	26.3	*Malassezia* sp.	invasive mycosis of the skin
8	biopsy from liver	negative	negative	*Rhizomucor* sp.	34.6	*Rhizomucor pusillus*	clinical suspicion of mucormycosis
9	biopsy from ethmoid	positive	negative	*Scedosporium* sp.	26.1	*Scedosporium* sp.	ethmoidectomy after chronic sinuitis, invasive mycosis
10	cornea	negative	negative	negative	34.3	*Fusarium* sp.	keratitis, clinical suspicion of invasive mycosis
11	biopsy from the sinus	positive	negative	negative	31.8	*Aspergillus* sp.	chronic fungal sinusitis, clinical suspicion of invasive aspergillosis
12	cornea	negative	negative	negative	31.1	*Fusarium* sp.	keratitis, clinical suspicion of invasive mycosis
13	blood (EDTA)	nd	negative	negative	30.0	*Rhizomucor pusillus*	clinical suspicion of invasive mycosis

Among the 151 analysed specimens, we did not observe any culture positive and PCR negative samples, but 13 specimens from “confirmed fungal infection” cases were 18S rDNA RT-PCR positive and culture negative (Table 4). Therefore, sensitivity of the 18S rDNA RT-PCR was higher compared to culture, while specificity was equal among both methods using a composite diagnostic measure combining clinical and microbiological data as gold standard. Overall, we found a concordance of 91% between 18S rDNA RT-PCR and culture results for the diagnosis of fungal infections (Table 3).

## Discussion

Early diagnosis of fungal infection is critical for effective treatment. There are many impediments to classical diagnostic methods like culture and microscopy such as increasing diversity of fungal pathogens, diminishing number of experienced clinical mycologists, costs and long time to result. Molecular methods offer the opportunity for determining the presence and diversity of fungi in clinical specimens and can readily be integrated in strategies for full lab automation.

In this study, a set of well characterized clinical samples was used to evaluate a molecular based algorithm for fast and reliable fungal detection and identification. The proposed diagnostic workflow includes automated DNA extraction and high throughput screening of clinical specimens by 18S rDNA RT-PCR (batches of 24 clinical samples can be analyzed per run). This procedure offers rapid diagnosis of fungal infections (<4 hours to identify a clinical sample as negative and <8 hours to obtain species identification by 18S rDNA sequencing). The diagnostic workflow does not require time consuming semi-nested PCRs and CleanGel analysis for visualization of ITS PCR products. An 18S rDNA specific fungal probe is used in the RT-PCR, and amplification products can be sequenced directly if a Ct value is obtained in RT-PCR. This is particularly useful as the majority of specimens that have been sent to our diagnostic laboratory over the last years (2013 to 2017) tested negative by culture and/or ITS PCR. Rapidly receiving a negative test result may assist the treating physicians in their decision to stop antifungal therapy that can have severe adverse effects (e.g. hepatoxicity and nephrotoxicity) for the patient^14^.

Based on species discrimination across the fungal kingdom, ITS is generally superior to the 18S rRNA gene, allowing higher phylogenetic resolution regarding species identification of some genera, like *Candida* and *Aspergillus*. However, the increased phylogenetic resolution is accompanied by lower sensitivity in fungal detection (12 clinically confirmed fungal infection cases missed by ITS PCR). In fact, the FungiQuant^®^ primer/probe set used in this study was designed in silico to completely cover the 18S rDNA target region of most fungal phyla (Saccharomycotina, Taphrinomycotina, Pezizomycotina, Mucoromycotina, Pucciniomycotina, Agaricomycotina)^15^. This allows sensitive detection of a diverse range of fungal species and high amplification efficiencies.

Empiric treatment of fungal infections like aspergillosis, cryptococcosis, mucormycosis, and invasive candida infections normally doesn’t require species specific identification^16^. A more crucial factor is the rapidity with which guided antifungal treatment can be administered. If phylogenetic resolution of the 18S rDNA gene is not sufficient for species level identification, a second PCR that targets other fungal marker genes^17^ can be additionally used in a routine diagnostic testing algorithm.

Overall, 18S rDNA RT-PCR amplification showed higher efficiency and revealed better results than ITS PCR. ITS sequencing often revealed ambigious sequences that could not be interpreted. Therefore, 117 sequencing reactions were necessary to obtain 78 interpretable Sanger electropherograms. In contrast, sequencing of 18S rDNA always revealed interpretable electropherograms, which led to reduced workload and costs. 18S rDNA sequencing also showed higher sensitivity than ITS PCR as ITS PCR did not detect fungal pathogens in 12 clinical specimens. ITS PCR did not identify *Rhizomucor* sp. and *Rhizopus* sp. in 4 clinical specimens, which is problematic as mucormycosis is a severe disease associated with a high mortality rate^18^. Therefore, sensitive detection of mucorales is a necessary requirement for molecular assays. ITS PCR failed to provide a species identification in one case of *Pneumocystis jirovecii* associated pneumonia. *Pneumocystis jirovecii* causes life-threatening infection, mainly in immunocompromised patients^19^. Lower diagnostic sensitivity of the ITS PCR may also explain the negative results from paraffin sections (N = 5). Though fixatives do not physically degrade nucleic acids *per se*, they reduce the amplifiable quantity of available DNA in clinical specimens^20^.

Two clinical specimens were ITS PCR positive and 18S rDNA RT-PCR negative. Both specimens showed a Ct value > 36 in the 18S rDNA RT-PCR indicating low amounts of available DNA in the clinical specimen. Indeed, review of the original silver stained CleanGel showed rather weak PCR bands in the CleanGel. If the DNA yield is low in the clinical specimen, prolonged freezing and thawing of genomic DNA may lead to progressive DNA degradation^21^, and it may potentially explain the inability to identify the fungal pathogens by 18S rDNA sequencing.

One requirement for using molecular based fungal identification in an accredited diagnostic laboratory is standardized 18S rDNA sequence identification. Generally, 18S rDNA sequence data can be used to search public databases, such as GenBank, using the web-based BLASTn algorithm. Unfortunately, the uncurated nature of Genbank has always been problematic for fungal identification, and therefore sequence analysis should be performed with extreme caution owing to the high frequency of erroneous deposits^22^. This has led to the development of both commercially and publicly curated, closed databases like SmartGene (SmartGene GmbH, Lausanne, Switzerland), RipSeq (Pathogenomix, Santa Cruz, USA) or the SILVA ribosomal RNA database project^23^. We used two SmartGene databases, the ITS SmartGene IDNS database that contains at present 413,375 curated entries (from approximately 777,690 available ITS sequences in Genbank) and the 18S SmartGene IDNS database that contains at present 47,592 curated entries (from approximately 230,000 available “true” 18S sequences in Genbank). Only sequences of high quality (e.g. good coverage, well annotated, only a few Ns and gaps in the sequence) are accepted in these SmartGene IDNS databases to allow standardized, reproducible and accurate identification of fungal pathogens.

## Conclusions

The goal of this study was to evaluate a diagnostic workflow for fungal identification using automated DNA extraction and 18S rDNA RT-PCR, followed by SANGER sequencing and species identification using the SmartGene IDNS database. We found high concordance between 18S rDNA RT-PCR, ITS PCR and culture, respectively for the diagnosis of fungal infections. Compared to ITS PCR and culture, 18S rDNA RT-PCR has the advantages of higher sensitivity, faster processing (<1 working day) and prospect for a high degree of laboratory automation.

## Materials and Methods

### Study design, clinical specimens and medical record review

Clinical samples were sent to the Institute of Medical Microbiology from secondary and tertiary hospitals from the Zurich metropolitan area for fungal identification by culture and/or ITS PCR. The specimens were mainly from normally sterile body sites (fresh biopsies (N = 100), punctates and deep wound secretions (N = 66), paraffin sections (N = 17), whole blood (N = 9), CSF (N = 7), bone marrow (N = 3), bones (N = 3)) but also from the eye and from the ear-nose-throat area (respiratory materials (N = 28) such as bronchoalveolar lavage, respiratory swabs, sputum, tracheal, broncheal and nasopharyngeal secretions). The retrospective study included 233 clinical specimens from unique patients analysed between 2013 and 2017 at our ISO accredited diagnostic laboratory. Diagnosis of fungal infection was done combining underlying disease and disease history of the patient, clinical course of disease and interventions, clinical signs and symptoms of inflammation, additional diagnostics if available (such as radiology and pathology reports), and microbiological findings (microscopy, culture, PCR results, detection of bacterial or viral pathogens) including detailed consultations with the treating physicians. Therefore, all included patient samples in the retrospective study could be categorized as “confirmed fungal infection” or “no fungal infection”.

### Phenotypic methods

Clinical specimens were analysed by culture methods and microscopy for the presence of fungi as described previously^24–26^. Briefly, specimens were cultured on general mycology media (Sabouraud dextrose agar containing gentamicin and chloramphenicol, and brain heart infusion (BHI) agar; Becton Dickinson AG, Allschwil, Switzerland) and on fungal selective media (Chromagar, and Mycosel; Becton Dickinson AG) for a maximum of 3 weeks at 25 °C and were regularly examined for growth by eye. Subcultures for identification were done as follows: (i) *Aspergillus* spp. on malt yeast agar^6^ at 25, 35, and 42 °C; (ii) mucorales on potato carrot agar at 25, 37, 40, 45, 50, and 56 °C; (iii) all other molds on Sabouraud dextrose agar containing gentamicin and chloramphenicol at 25 and 35 °C and on Mycosel and potato carrot agar, both at 25 °C (temperatures as routinely used in our clinical laboratory). Phenotypic identification was based on macro- and micro-morphological criteria^26^.

### Genotypic methods

DNA from clinical specimens was extracted on the EZ1 Advanced XL (QIAGEN, Hombrechtikon, Switzerland) using the EZ1 DNA Tissue Kit (QIAGEN, Hombrechtikon, Switzerland), following the manufacturer’s instructions. A process control was included (i.e. water sample) in each analysis to exclude fungal DNA contamination in the DNA extraction chemistry, buffers and PCR reagents.

PCR amplification of the ITS region was performed using ITS1 and ITS4^12^ as described previously^9^. Amplification products were visualized by polyacrylamide gel electrophoresis (CleanGel 10% 52S, ETC GmbH) combined with silver staining. Subsequently, a semi-nested PCR was performed and ITS PCR products were purified with the QIAquick PCR purification kit (Qiagen, Hombrechtikon, Switzerland). ITS PCR products were sequenced with primer ITS4 and ITS3^12^ using the BigDye kit (Life Technologies, Zug, Switzerland) and an automated DNA sequencer (ABI Prism 3130 Genetic Analyzer; Life Technologies, Zug, Switzerland). Fungal identification of ITS PCR products was done using the ITS SmartGene IDNS custom platform, following the identification guidelines from Ciardo *et al*.^24^.

DNA from the clinical specimens (N = 233) was retrospectively analysed by 18S rDNA RT-PCR. For the 18S rDNA RT-PCR, 5 µl of extracted DNA was added to a mixture consisting of 8.5 µl Roche water (Roche, Rotkreuz, Switzerland), 4 µl of a LightCycler^®^ DNA multiplex master mix (Roche), 0.5 µM of each FungiQuant^®^ primer and 0.25 µM of the FungiQuant^®^ probe^15^. Cycling parameters included an initial denaturation for 5 min at 95 °C, followed by 45 cycles of 5 sec at 95 °C, 15 sec at 60 °C and 15 sec at 72 °C. Subsequently, the 18S rDNA RT-PCR products were purified with the QIAquick PCR purification kit, and sequenced using the BigDye kit and an automated DNA sequencer (ABI Prism 3130-Genetic Analyzer; Life Technologies, Zug, Switzerland). Sanger electropherograms were visually examined (regularity of base spacing, distribution of peak heights, occurrence and height of minor background peaks). Any part of the electropherogram that showed irregularities (e.g. high background noise, irregular base spacing and peak height distributions) was excluded from the reported result. Accurate fungal identification was achieved by analysing sequences with a quality score >20 (“>Q20”) that covered at least 300 bp of the respective fungal 18S rRNA gene in the 18S SmartGene IDNS custom platform. Species and genus level identification was done following the criteria by Ciardo *et al*.^24^.

A major disadvantage of PCR in comparison to culture is the frequent contamination of reagents and materials with traces of fungal DNA^27^. We used three criteria to uncover environmental contamination and to strictly categorize samples as negative: (1) no distinct ITS PCR fragment in the CleanGel or no PCR amplification with a Ct value in the 18S rDNA RT-PCR, (2) fungal identification in a clinical specimen identical to contamination in the process control, (3) identification of a known environmental contaminant.

### Statistics

A synopsis of clinical findings (disease history, clinical picture) and laboratory results (microscopy, ITS PCR, culture) including consultation with the treating physician was used as gold standard to categorize patients into “clinically confirmed fungal infection” or “no fungal infection” cases. On the basis of this composite diagnostic measure, we used the 2 × 2 contingency table to calculate the agreement between culture, ITS PCR and 18S rDNA RT-PCR results^28,29^.

### Ethics statement

The study was conducted according to good laboratory practice and in accordance with the Declaration of Helsinki and national and institutional standards. The act on medical research involving human subjects does not apply to this study. In this study, solely extracted DNA from clinical specimens an anonymized health realted data were used, therefore no consent from the ethics committe was required.

### Availability of data

The datasets generated and analysed during the current study are available from the corresponding author on reasonable request.

## Electronic supplementary material

Supplementary Information
