# Supplementary material for: Molecular detection of fungal pathogens in clinical specimens by 18S rDNA high-throughput screening in comparison to ITS PCR and culture
Source: Sci Rep. 2018 May 3;8:6964. doi: 10.1038/s41598-018-25129-w (PMC5934447; doi:10.1038/s41598-018-25129-w)
Supplement: Supplementary file 1 — Supplementary Information [file 41598_2018_25129_MOESM1_ESM.docx]

**Molecular detection of fungal pathogens in clinical specimens by 18S rDNA high-throughput screening in comparison to ITS PCR and culture**

K. Wagner, B. Springer, V. P. Pires, P.M. Keller

| **Species** | **Count** |
| --- | --- |
| *Carassea connexa* | 1 |
| *Epichloe typhina* | 1 |
| *Funneliformis mosseae* | 7 |
| *Gymnoconia peckiana* | 1 |
| *Parastagonospora nodorum* | 1 |
| *Phakopsora pachyrhizi* | 1 |
| *Stereocaulon urceolatum* | 2 |

Table S1. All fungi that were identified in clinical specimens and assessed as contaminants.
